# Real-time atomistic observation of structural phase transformations in individual hafnia nanorods

**DOI:** 10.1038/ncomms15316

**Published:** 2017-05-12

**Authors:** Bethany M. Hudak, Sean W. Depner, Gregory R. Waetzig, Anjana Talapatra, Raymundo Arroyave, Sarbajit Banerjee, Beth S. Guiton

**Affiliations:** 1 Department of Chemistry, University of Kentucky, 505 Rose Street, Lexington, Kentucky 40506, USA; 2 Department of Chemistry, University at Buffalo, The State University of New York, Natural Sciences Complex, #359, Buffalo, New York 14260-3000, USA; 3 Department of Chemistry, Texas A&M University, 3255 TAMU, 580 Ross St, College Station, Texas 77843, USA; 4 Department of Materials Science and Engineering, Texas A&M University, 575 Ross St, College Station, Texas 77843, USA; 5 Materials Science and Technology Division, Oak Ridge National Laboratory, 1 Bethel Valley Rd, Oak Ridge, Tennessee 37831, USA

## Abstract

High-temperature phases of hafnium dioxide have exceptionally high dielectric constants and large bandgaps, but quenching them to room temperature remains a challenge. Scaling the bulk form to nanocrystals, while successful in stabilizing the tetragonal phase of isomorphous ZrO_2_, has produced nanorods with a twinned version of the room temperature monoclinic phase in HfO_2_. Here we use *in situ* heating in a scanning transmission electron microscope to observe the transformation of an HfO_2_ nanorod from monoclinic to tetragonal, with a transformation temperature suppressed by over 1000°C from bulk. When the nanorod is annealed, we observe with atomic-scale resolution the transformation from twinned-monoclinic to tetragonal, starting at a twin boundary and propagating via coherent transformation dislocation; the nanorod is reduced to hafnium on cooling. Unlike the bulk displacive transition, nanoscale size-confinement enables us to manipulate the transformation mechanism, and we observe discrete nucleation events and sigmoidal nucleation and growth kinetics.

## Abstract (web summary)

The high-temperature tetragonal phase of HfO_2_ is technologically useful but difficult to stabilize at room temperature. Here, the authors observe in real-time the transformation of a HfO_2_ nanorod from its room temperature to tetragonal phase, at 1000° less than its bulk temperature, suggesting that size confinement may kinetically trap this phase.

The continued miniaturization of device components such as metal oxide semiconductor field-effect transistors^1^,^2^,^3^,^4^ has inspired a major push to replace silicon dioxide with high-*κ* dielectrics as the gate material^1^,^2^,^3^,^4^,^5^. Increasing the dielectric constant of gate oxides is imperative for continued reduction of the gate thickness, without deleterious leakage currents typical of ultrathin SiO_2_ when it is reduced to the nanometre scale. The mixed ionic–covalent nature of the Hf–O bonds in HfO_2_ polymorphs contribute to a high Born effective charge tensor Z*, and the combination of large Z* with the presence of soft low-energy phonon modes yield large dielectric constants^6^. Since the *κ*-values of oxides tend in general to vary inversely with band gap, in oxide-based components there is often a trade-off between a sufficiently high *κ*-value and a sufficiently large band gap, and therefore not all high-*κ* materials are useable. Amorphous and nanocrystalline HfO_2_ and their SiO_2_ composites have already emerged as viable alternatives to SiO_2_ and have been scaled to manufacturing environments^6^. HfO_2_ has a high dielectric constant and a high bandgap and is furthermore stable on silicon^4^; in its room temperature monoclinic phase, however, HfO_2_ has a dielectric constant of only about 18. In contrast, the higher temperature tetragonal (stable above ∼1700 °C) and cubic (>2600 °C) phases have *κ*-values of 75 and 29, respectively^7^, and band gaps which reach a maximum value of 6 eV in the tetragonal phase^8^. There is great interest, therefore, in trapping the tetragonal phase of HfO_2_ at room temperature, which has thus far only been possible for extremely small crystallites that are <3.6 nm in size, or within defective matrices^9^,^10^,^11^,^12^.

A slight displacive distortion of the unit cells for the three polymorphs of HfO_2_ is necessary to undergo a diffusionless martensitic transition from monoclinic to tetragonal to cubic on heating^13^,^14^. Since only a small distortion (a 9° shear of the unit cell from monoclinic to tetragonal and a ca. 2.7% expansion of the cell volume)^15^ defines this structural phase transformation, preventing reversion back to the monoclinic phase on quenching the tetragonal phase to room temperature has proven a formidable challenge^13^,^14^. HfO_2_ is isomorphic to ZrO_2_ (refs 13, 14), and the two oxides have similar lattice parameters and the same space group in each phase. Insight may be gleaned, therefore, from examining the case of ZrO_2_, which has been successfully stabilized in the tetragonal phase at room temperature in nanocrystalline form^7^,^12^,^13^. This represents a suppression of the ZrO_2_ transition temperature by over 1000 °C from its bulk value of 1050 °C (refs 16, 17, 18, 19). Even for such classical martensitic transitions, however, the atomistic details of the transition and their specific size dependence remain to be elucidated.

The longer metal–oxygen bond length in the tetragonal (versus the monoclinic) phase is thought to be a key parameter that may be manipulated. The model developed by Grain and Campbell^20^ suggests that stretching of the M–O bond with simple thermal expansion is key to stabilizing the tetragonal phase above the bulk phase transition temperature, explaining the large discrepancy between the transformation temperatures of ZrO_2_ and HfO_2_ (refs 13, 20); the idea is simply that the Zr–O bond is longer than the Hf–O bond, and results in a correspondingly lower transition temperature of 1050 °C, in comparison to ∼1700 °C in HfO_2_. Following this logic, methods to effectively stretch this bond without relying on thermal expansion, such as working at reduced pressure, or introducing strain, or cation dopants^7^,^21^,^22^, could also be effective. In the latter case the resulting oxygen vacancies stabilize the tetragonal and cubic ZrO_2_ phases, although with the deleterious side effect of introducing charge traps, increasing ionic conductivity, and disrupting the dielectric properties of the material.

Tetragonal ZrO_2_ is stabilized via scaling the crystal to finite size^23^,^24^. In these crystals the surface energies, and the increased surface-to-volume ratio, successfully stabilizes nanocrystalline ZrO_2_ in the desired phase^25^. Interestingly, in each of the above approaches, the goal is to manipulate the structure such that the tetragonal phase is the thermodynamically stable phase at room temperature. An alternate approach would be to manipulate the mechanism by which the phase transition proceeds, so as to engineer a material in which the tetragonal phase may be trapped kinetically; to our knowledge this has not yet been attempted.

Similar approaches to the stabilization of ZrO_2_ have been attempted for HfO_2_, with mixed results. At atmospheric pressure, heating the pure material will induce a transformation from monoclinic to tetragonal at ∼1700 °C. Chemical doping has also been employed, although the resulting dielectric constant has yet to be fully determined^26^,^27^,^28^,^29^,^30^. Stabilizing tetragonal HfO_2_ through size-confinement is more difficult; in previous work it has been shown that when scaled to finite size, HfO_2_ will remain in the monoclinic phase down to the few-nanometre scale, and contains multiple twin boundaries (giving a nano-scale barcode appearance)^31^. Temperature-dependent X-ray diffraction (XRD) of these monoclinic HfO_2_ nanorods shows the monoclinic to tetragonal phase transformation commencing at 400 °C (ref. 31). Ensemble measurements are, however, unable to capture the richness of the modified phase diagrams accessible at ultra-small dimensions. The interplay between deformation, creation of twin boundaries, bond stretching and phase transformations has thus remained unexplored even for the classical displacive transition characterized by this simple binary oxide system. Indeed, despite considerable attempts to map energy landscapes^18^,^19^, much of the work in this area remains theoretical, and there exists no precedent for real-space and real-time examination of the phase transformation at the atomic scale.

Here, we use *in situ* heating in a scanning transmission electron microscope (STEM) to observe directly the phase change of a single hafnia nanorod. The transformation pathway of the nanorod from twinned-monoclinic to tetragonal is revealed with atomic-scale resolution, with nucleation at a twin boundary, followed by propagation of the tetragonal phase via a coherent transformation dislocation. Interestingly, size-confinement enables us to manipulate the transformation mechanism such that discrete nucleation events and conventional sigmoidal nucleation and growth kinetics are observed (attributed in part to plane-by-plane motion of the transformation dislocation), rather than the expected displacive transition, which (in the bulk) prevents quenching of the tetragonal phase.

## Results

### Atomic resolution imaging of HfO_2_ nanorods

**Figure 1:**
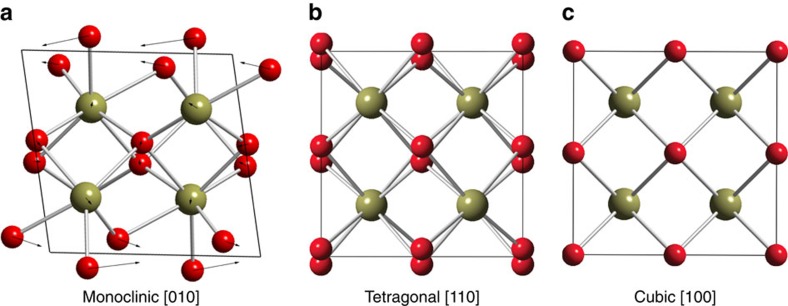
Crystal structures of HfO_2_ polymorphs. The unit cell of three phases of HfO_2_, showing the small atomic displacements, and preservation of the coordination environments, during transformation between the phases. The monoclinic to tetragonal (**a**,**b**) and tetragonal to cubic (**b**,**c**) transitions occur at ∼1700 and ∼2600 °C, respectively, at atmospheric pressure. Arrows in (**a**) show displacements necessary to transform to the tetragonal phase (**b**). Hafnium atoms are shown as olive spheres, whereas oxygen atoms are shown as red spheres.

**Figure 2:**
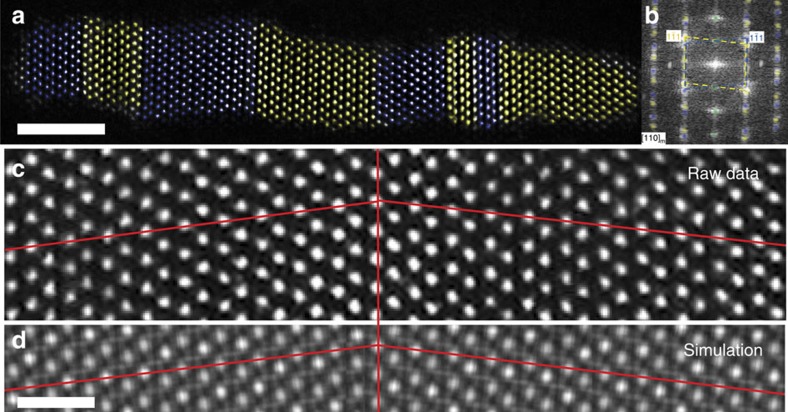
*Z*-contrast image of twinned monoclinic nanorod. (**a**) False-coloured HAADF image of a hafnia nanorod with twin domains imaged in the [110] zone coloured yellow and blue. Scale bar, 2 nm. (**b**) FFT of nanorod with twin domain spots masked in yellow and blue. (**c**,**d**) Magnified image and simulation, respectively, of a single twin boundary. The 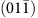 plane is mirrored about the (100) plane. The simulation provides a qualitative view of the expected contrast and is not intended to demonstrate quantitative contrast matching. Scale bar, 5 Å.

High aspect ratio, monoclinic hafnia nanorods were grown via a non-hydrolytic sol–gel synthesis, as described previously^31^. Unit cells for the three polymorphs of HfO_2_ are shown in Fig. 1, illustrating the slight displacive distortions necessary to undergo a diffusionless martensitic transition from monoclinic to tetragonal to cubic on heating^13^,^14^. Figure 2 shows high angle annular dark field (HAADF) ‘*Z*-contrast' imaging of the nanorod before heating, with accompanying simulated image (calculated with QSTEM software)^32^. The rods are monoclinic with multiple twin boundaries occurring along the (100) planes. These twins are believed to form to accommodate shear strain during the tetragonal to monoclinic phase transformation upon cooling during synthesis^31^. Upon increasing the length of the nanocrystals while keeping the diameter constant, the number of twins are monotonically increased^31^. The stabilization of such ferroelastic domains and coherent twin boundaries has previously been examined by dark-field TEM studies^31^. The nanorods represent excellent model systems since defects tend to migrate to surfaces and are annealed out during hot colloidal synthesis^33^,^34^. Indeed, no cation vacancies can be observed through atomic resolution imaging. The coherent twin boundaries visible in Fig. 2 are the primary imperfections discernible in these crystals. Measurements of bulk samples or even ensemble measurements of nanostructures furthermore cannot capture specific nucleation events given the large nucleation volumes and the facile propagation of transformations across the 3D material akin to twinning dislocations during transformation twinning. The dimensionally confined nanorods thus represent ideal model systems for examining discrete nucleation events and the propagation of the nucleated phase.

### Monoclinic to tetragonal transition of an HfO_2_ nanorod

**Figure 3:**
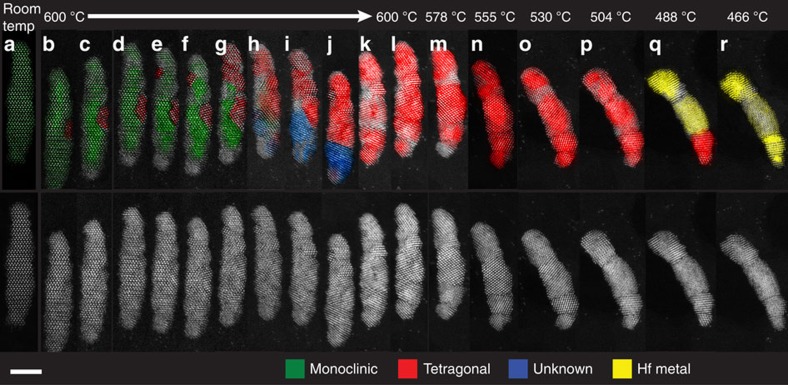
Structural phase transformation of a single hafnia nanorod heated in the STEM. Top: False-coloured HAADF images highlighting the structural phases present in each frame. (**a**) Before annealing, the nanorod is monoclinic. (**b**–**l**) Over a period of about 40 min the nanowire converts completely from single-crystalline twinned monoclinic to polycrystalline tetragonal hafnia with retention of the overall morphology. The transformation is nucleated at a surface defect that extends into a twin boundary. The nucleated tetragonal domain advances one discrete lattice plane at a time across two distinct twin boundaries, eliminating the boundaries in the process. Conservation of a one-to-one lattice correspondence across the phase boundary between advancing tetragonal and receding monoclinic phases suggests the propagation of a transformation dislocation. (**m**–**r**) Upon cooling, the nanorod is reduced from tetragonal hafnia to hafnium metal. Scale bar, 5 nm.

In Fig. 3 we show an individual nanorod as it undergoes a structural change during heating *in situ* in the STEM (Supplementary Movie 1). The nanorod in this figure was stepped from room temperature to 600 °C at a reported rate of 10^6^ °C s^−1^ and held at this temperature for 1 h; this temperature was chosen in order to target the midpoint of the mixed phase region observed with *in situ* heating XRD^31^. The sample holder allows for rapid and homogeneous temperature equilibration and allows for the target temperature to be reached within a few seconds^35^. Such a setup thus allows for isothermal kinetics to be mapped without the substantial thermal gradients that can result in cracking of bulk samples. Other wires heated under similar conditions and showing similar structural changes are described in Supplementary Figs 1 and 2, though in several of these cases the crystallites within each wire are too small to clearly identify their crystal structures during transformation.

The high resolution of the images collected allows us to observe the structural changes directly. To estimate the volume of each phase contained within the wire in Fig. 3 as a function of time, we used a fast Fourier transform (FFT) approach. For each frame, an FFT of the nanorod was acquired, and the spots in this pattern were identified to belong to one or more specific hafnium oxide phase. So identified, the spots were masked, and an inverse FFT (IFFT) was used to regenerate those parts of the image containing the selected phase. Using this technique, we are able to map out the different phases (and their crystallographic zone axes) present in the nanorod over time, directly observing the phase transformation as it occurred. An unknown phase appears transiently (Fig. 3, blue regions), and is neither monoclinic nor tetragonal, but is most similar to the orthorhombic hafnia phase^19^, although it cannot be identified unambiguously. We speculate that the orthorhombic hafnia (or a distorted version of it) might provide a transition pathway for the monoclinic to tetragonal phase transformation for some orientation relationships but a direct monoclinic–tetragonal transformation appears to be the predominantly observed pathway as further discussed below.

**Figure 4:**
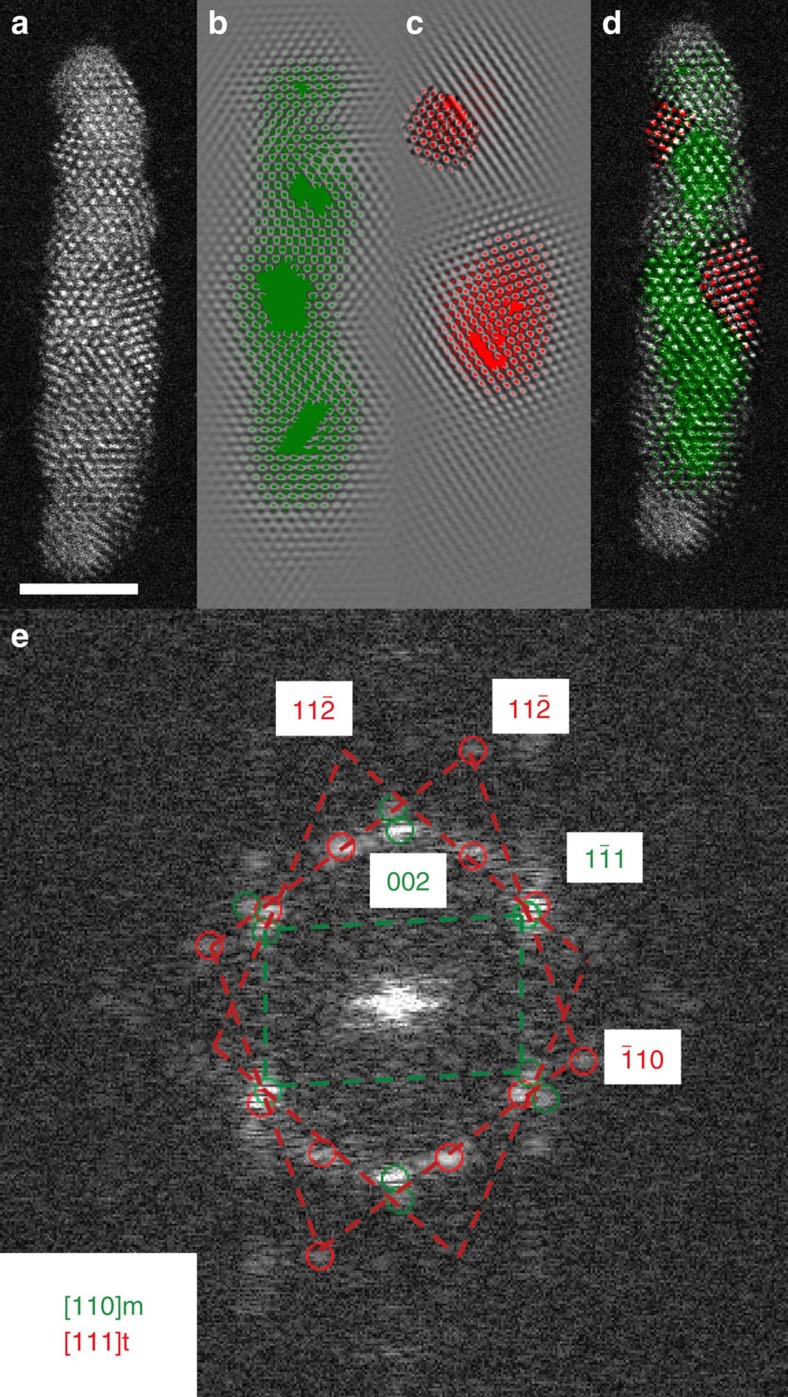
A single frame captured during nanorod heating. (**a**) HAADF image, Scale bar, 5 nm. (**b**) False-coloured IFFT of the monoclinic phase of HfO_2_. (**c**) False-coloured IFFT of the tetragonal phase of HfO_2_. (**d**) Data from (**b**–**c**) overlaid onto (**a**). (**e**) FFT of (**a**) with monoclinic and tetragonal spots circled.

Figure 4 demonstrates this analytical approach for Fig. 3f, chosen as an example. In this panel, collected shortly after two tetragonal regions have nucleated, the FFT/IFFT approach reveals a single-crystalline region of the unchanged monoclinic [110] zone, and two small regions of tetragonal HfO_2_, both oriented along the tetragonal [111] axis (rotated around [111] with respect to one another). A similar analysis was performed on the FFT of each captured frame, in each case identifying the phases present, and their orientations. Supplementary Fig. 3 shows representative FFT analyses of Fig. 3a,f,i,n,r, and a more detailed description of the phase identification procedure is outlined in Supplementary Methods.

Figure 3 shows clearly that upon heating to 600 °C, nucleation of the tetragonal phase is visible after 20 min. The phase change appears to be defect-nucleated, beginning at a surface defect that extends into one of the previously identified twin boundaries. Using the FFT analysis (Fig. 4) and atomic resolution imaging, we are able to identify the emerging phase as tetragonal HfO_2_, and over a period of about 40 min the nanowire converts completely from single-crystalline twinned monoclinic to polycrystalline tetragonal hafnia, preserving much of the nanorod's overall morphology. Notably, no migration of oxygen vacancies is observed in this regime.

The tetragonal phase must first be nucleated to initiate the phase transformation and it is clear that the nucleation event in Fig. 3 is associated with the twin planes, which correspond to a relatively higher free energy starting point as compared to the interior of an untwinned monoclinic domain. Indeed, Supplementary Fig. 2 provides another example of nucleation of the tetragonal phase in close proximity to a coherent twin boundary at the bottom end of a particle. Both of these images permit examination of discrete nucleation events and the identification of the nucleation sites, which would otherwise not be possible in extended solids with a large free volume available for nucleating phase transformations. While nucleation phenomena have not been extensively studied in these systems, examination of the displacive monoclinic to tetragonal phase transition of a related binary oxide, VO_2_, suggests a pronounced hysteresis between the forward and reverse transitions since the forward monoclinic→tetragonal transition can readily be nucleated at M1/M2 (two distinct monoclinic polymorphs) phase boundaries and at twin boundaries, whereas the reverse tetragonal→monoclinic transition has to be nucleated at point defects in the absence of high-energy twin planes^36^,^37^. Even within these systems, nucleation phenomena have been surmised from the behaviour of strained and defective materials and direct observation remains elusive. In these materials, dimensional confinement reduces the volume density of point defects and thus results in kinetic stabilization of high-temperature phases^38^.

Close inspection of the FFT in Fig. 4 shows close to overlapping pairs of monoclinic (green) and tetragonal (red) spots, indicating sets of parallel planes with similar *d*-spacings from the two phases, suggesting likely candidates for the monoclinic–tetragonal phase boundary. One such pair are {011}_t_/{111}_m_, which forms the lower bounding interface of the larger of the two tetragonal regions, presumably at the location of a transformation dislocation^39^. Supplementary Fig. 4 highlights the growth of this tetragonal domain during the annealing process. As the dislocation migrates, the {011}_t_/{111}_m_ phase boundary (highlighted in blue) grows (from 0 to ∼840 s), until it changes orientation over the period of ∼840 to 1320, s. An atomistic model of the {011}_t_/{111}_m_ interface (Supplementary Fig. 5) illustrates that this boundary is coherent, consistent with a model of migrating transformation dislocation.

Considering Fig. 3a–d, a clear faceted crystallographic relationship is observable across the phase boundary (monoclinic in green and tetragonal in red) with (111) and (111)* planes of the monoclinic phase interfaced with (011) planes of the tetragonal phase. The incipient tetragonal domain advances simultaneously across two distinct twin boundaries that are separated by three lattice planes, eliminating the boundaries in the process since the higher symmetry of the tetragonal phase does not permit twinning. The energetic costs for propagation of the tetragonal phase and for having a phase boundary are thus clearly offset in part by elimination of the interfacial energy at the twinned interface. Notably, the propagation of the tetragonal phase occurs one discrete lattice plane at a time (contrasting Fig. 3c,d,f) with conservation of a one-to-one lattice correspondence across the phase boundary (illustrated in Supplementary Fig. 5); the conservative motion and lattice correspondence suggest that deformation is induced via a transformation dislocation^39^,^40^. By Fig. 3h, the twin planes have been eliminated and conservative motion of this domain can no longer be continued without a change of lattice plane. The strain resulting from partial deformation of the lattice likely contributes to further nucleation events.

### An evaluation of the energetics of the phase transformation

**Figure 5:**
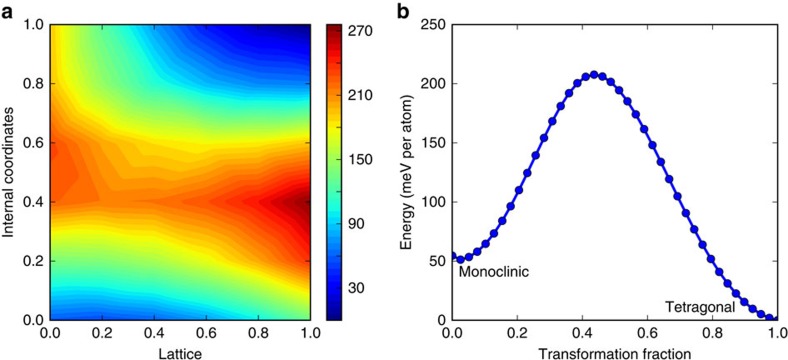
Energy landscape and activation energy barrier for HfO_2_. (**a**) Energy landscape of the monoclinic to tetragonal phase transformation of HfO_2_ (without twin planes) calculated using two parameters: (i) change in lattice and (ii) change in internal coordinates. Here, (0,0) corresponds to the rutile/tetragonal phase and (1,1) corresponds to the monoclinic phase. Calculations were carried out at grid points corresponding to a 9 × 9 grid, which were then interpolated to construct the landscape. A steepest descent algorithm was employed to find the minimum energy path (MEP) of the transformation. Colour bar in meV per atom. (**b**) Energy barrier for the monoclinic to tetragonal phase transformation.

Figure 3 indicates that the transition temperature is depressed by over 1000 °C as compared to the bulk and exhibits kinetics that are strongly atypical for a displacive transition, suggesting a pronounced modification of both thermodynamic stabilities and activation barriers. Considering the former, the change in free energy (Δ*G*) across the M→T transformation can be written as


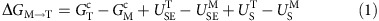


where 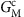 and 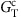 values are the chemical free energies of the monoclinic and tetragonal phases (dependent on temperature), respectively; the 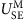 and 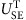 values are the strain energies for the monoclinic and tetragonal phases, and the 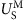 and 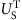 values are the surface free energies for the monoclinic and tetragonal phases^41^. The calculated temperature dependencies for the bulk free energy, enthalpy, entropy and specific heat capacity, as determined from density functional theory (DFT) calculations, are shown in Supplementary Fig. 6. At the transition temperature, thermodynamics requires 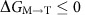. At a temperature of 600 °C, the chemical free energy difference, 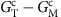 for the M→T transformation is expected to be strongly positive and as per the energetics of the bulk phase diagram (Supplementary Fig. 6), the monoclinic phase ought to remain stable in preference to the tetragonal phase. This implies that the differentials in strain energy and surface free energy underpin the observed strong depression of the transition temperature. Supplementary Fig. 7 identifies the surface planes stabilized for the monoclinic and tetragonal phases of HfO_2_. With the exception of a few planes in immediate proximity of the twin planes, the side surfaces of monoclinic HfO_2_ are {110} planes with {001} planes binding the ends. In addition to these planes, transformation to the tetragonal phase further exposes {010}, {101} and {112} planes. DFT calculations of surface energies by Ramprasad and co-workers^19^ suggest that the surface energies of {110} and {001} planes are substantially higher for the monoclinic phase (1.38 and 1.51 J m^−2^, respectively) as compared to the values for the tetragonal phase (1.08 and 1.21 J m^−2^, respectively). In other words, as also observed for ZrO_2_ (refs 9, 42), the tetragonal phase of HfO_2_ has a substantially lower surface energy and thus 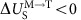. This term is furthermore expected to be strongly size-dependent as per:


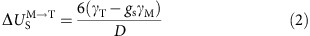


where the *γ*_T_ and *γ*_M_ terms represent the interfacial surface energies of the tetragonal and monoclinic phases, respectively, *D* is the diameter of the particle and *g*_s_=*A*_M_/*A*_T_ the ratio of the interfacial surface areas with the subscripts denoting the values for the monoclinic and tetragonal polymorphs^41^. Clearly Equations (1) and (2) together predict that the transition temperature will be directly proportional to the diameter of the particle. Ramprasad and co-workers have predicted extended phase stabilities of the tetragonal phase at finite dimensions as indeed observed here and for ultra-small nanocrystals wherein the tetragonal phase can be stabilized at room temperature^9^,^19^ based on surface energy considerations. While such surface energy effects have been noted for other systems as well^42^, the effect of twinning is no less important. The plastic deformation that induces the twin planes shown in Fig. 2 partially alleviates the strain induced upon deformation of a tetragonal particle (the T→M transition) during synthesis. As a result of the energy stored in these deformations that can in turn be dissipated during the M→T transformation, the differential in strain energy 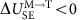 further contributes to driving down the transition temperature. Lange^41^ has developed a size-dependent expression for the energy of the twin surface per unit volume that equates to 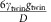 where *γ*_twin_ is the twinning energy per unit area, *D* is the particle diameter and *g*_twin_ is a dimensionless quantity that can be expressed as 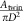, where *A*_twin_ is the total area of the twin boundaries. Figure 5 plots the energy landscape and activation energy barrier calculated for the transformation from monoclinic to tetragonal HfO_2_ as deduced for bulk HfO_2_ using DFT calculations. The activation energy barrier for the transformation is deduced to be ca. 207.63 meV atom^−1^. Twinned regions correspond to relatively high free energy regions of the energy landscape (raised by the interfacial energy of the twin boundaries). Consequently, the activation energy for transformations initiated at twinned domains will be lower. Indeed, Fig. 3 and Supplementary Fig. 2 both suggest that these regions serve as the nucleation sites for initiating the phase transformation. In other words, the high local density of twins and the small particle size both contribute to the >1000 °C depression of the transition temperature from the bulk values and the strongly modified phase equilibrium observed at the nanoscale.

### Kinetics of the phase transformation

**Figure 6:**
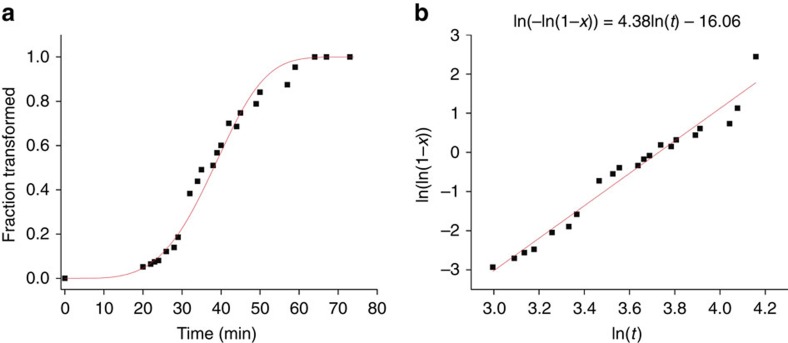
JMAK kinetics plots. (**a**) Fraction of transformed phase versus time, displaying the sigmoidal curve characteristic of nucleation and growth phase transformation kinetics. (**b**) Linear plot to determine the value of *n* (slope).

Interestingly the kinetics of this phase transformation are also not typical of the martensitic and athermal processes known to occur in bulk HfO_2_^13^,^31^,^43^,^44^. In the bulk, the transformation mechanism is a diffusionless process, in which bond angles and distances rearrange without disrupting atomic connectivity, and this mechanism should therefore produce an abrupt change in lattice parameters that propagates across the entire crystal^44^. Furthermore, the athermal nature of the bulk process suggests that a change in temperature is required for transformation^13^. Conversely to bulk observations, we observe a relatively slow transformation under isothermal conditions with the rate determining step apparently being the propagation of the transformation dislocation from one lattice plane to another. To investigate the transformation kinetics further we quantified the fraction of the nanorod that had transformed from the monoclinic to the tetragonal phase and applied the Johnson–Mehl–Avrami–Kolmogoroff (JMAK) model to infer the mechanism of phase transformation kinetics. The JMAK model describes thermally activated nucleation and growth kinetics of phase transformations:


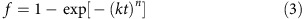


where *f* is the fraction of transformed volume, *t* is time, *k* is a constant and *n* is an integer or half-integer. The exponent *n* is the Avrami exponent, and describes the rate and geometry of nucleation and growth^45^,^46^,^47^. Within this model, *n* can be expressed as


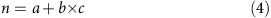


where *a* is the time-dependent nucleation rate, *b* is the dimensionality of the growing crystals and *c* is the growth rate^47^. The value of *a* gives a measure of nucleation rate; when *a*=0, no nucleation is observed, whereas an *a* value of 1 suggests a constant nucleation rate. When 0<*a*<1, the nucleation rate is decreasing, and *a*>1 suggests an increasing nucleation rate. The value of *b* is typically either 1, 2 or 3, corresponding to the dimensionality of phase growth, and *c* is either 1 or 0.5, corresponding to volume diffusion controlled growth and interface movement controlled growth, respectively^47^. When the fraction of the transformed phase is plotted versus time, it produces a sigmoidal curve characteristic of nucleation and growth kinetics described by the JMAK model, as shown in Fig. 6a. The data can be further plotted as (ln(1−*x*)) versus ln(*t*), where *x* is the fraction of phase transformed, which produces a straight line with slope equal to *n* and a *y*-intercept of *k* (Fig. 6b). The plot in Fig. 6b yields *n*=4.38. Because we directly observe interface movement growth (*c*=0.5) in Fig. 3 and Supplementary Fig. 4, and we know the nanorods are one-dimensional and can therefore assume one-dimensional phase growth (*b*=1), a value *a*>1 can be deduced, indicating an accelerating and autocatalytic nucleation rate. This is consistent with our observation, and the data strongly suggest that these nanorods undergo classical autocatalytic nucleation and growth kinetics. Based on Fig. 3, the observed motion of the transformation dislocation is responsible for the initial sigmoidal state and likely constitutes the rate-limiting step of this transformation. Importantly, this implies that through size-confinement, we are able to alter the phase transformation kinetics from a diffusionless mechanism to a nucleation and growth mechanism, potentially allowing for the high-temperature tetragonal phase to be quenched to room temperature if an activation energy barrier of sufficient magnitude can be induced.

### Reduction to Hf metal upon cooling

**Figure 7:**
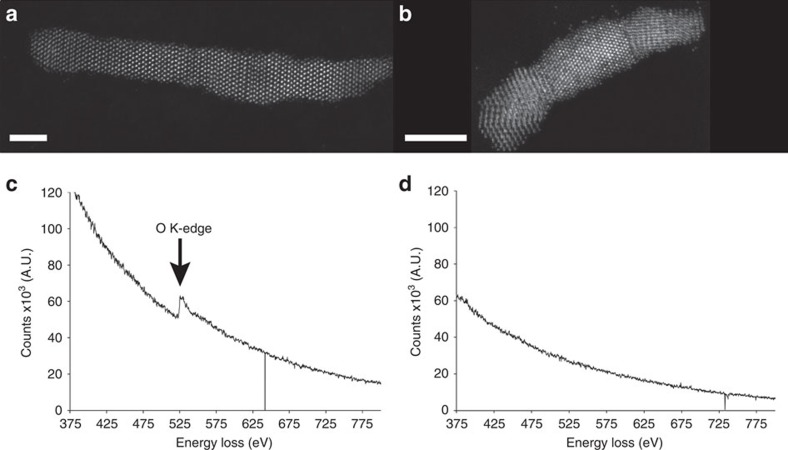
Electron energy loss spectroscopy from two hafnia nanorods before and after heating. (**a**,**c**) HAADF image and EEL spectrum, respectively, of a nanorod before heating. The oxygen K-edge is present. (**b**,**d**) HAADF image and EEL spectrum, respectively, of a different wire after heating and cooling. No oxygen K-edge is present, indicating the reduction of the rod to hafnium metal. Both scale bars are 5 nm.

Once the monoclinic to tetragonal transformation was complete, we cooled the nanorod over a period of 160 min (at 0.015 °C s^−1^) to 456 °C (Supplementary Movie 2), to observe any possible structural rearrangements and their kinetics. Interestingly, rather than forming a metastable tetragonal hafnia, or returning to a monoclinic hafnia phase, the Hf–O bonds in the nanorods are seen to distort, and the nanorod then proceeds to lose oxygen, reducing abruptly to hafnium metal at 530 °C, as evidenced by electron energy loss spectroscopy displayed in Fig. 7.

The distortion of the tetragonal phase on cooling is most likely due to oxygen loss, driven by the low oxygen fugacity in the microscope column, and can be measured in our nanorod using the 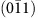 and 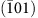 lattice spacings (Supplementary Fig. 8). Indeed, temperature variant XRD measurements performed by heating the samples up to 900 °C and then back down to room temperature do not show any indications of oxygen loss or stabilization of metallic Hf (Supplementary Fig. 9). FFTs were acquired from frames of the tetragonal nanorods during cooling, and the distances to the 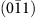 and 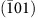 spots were measured. The *d*-spacing values change by 5% from the native lattice spacings—expanding in the 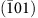 direction and contracting in the 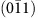 direction—before the crystal undergoes transformation to hafnium metal. This gives a quantitative measure of the extent of hafnium reduction which may be tolerated before the zero valent phase is stabilized.

## Discussion

The reduction of HfO_2_ to hafnium metal is likely driven by the high vacuum, low oxygen partial pressure of the STEM column, which is in the 10^−8^–10^−10^ Torr range during operation. Although beam damage of the rod cannot be completely ruled out, the zero valent hafnium metal phase may well be the thermodynamically stable phase for crystals of this reduced size (note that the transformation yields tetragonal crystallites that are further reduced in size as compared to the monoclinic nanorod) and under low oxygen fugacities. This would be in line with the work by Navrotsky^42^ and others, showing that phase diagrams constructed for binary transition metal oxides, as a function of both crystal size and low oxygen pressure, typically enter a small-size regime at low pressure in which the metallic phase is more stable than one or more of the binary oxide phases, which under these conditions are now only metastable. As an alternative mechanism, for an analogous M→T transition in VO_2_, Wu and colleagues^37^ have suggested that while as observed here, nanometre-sized twin walls can nucleate the M→T transition, the absence of twinning in the T phase requires that the reverse transition be nucleated at point defects, which are greatly reduced upon dimensional reduction to ultra-small volumes, resulting in substantial supercooling of the high-temperature phase. The large activation energy for nucleation of the M phase upon elimination of twin boundaries may under conditions of low oxygen fugacity result in preferential stabilization of metallic Hf.

Using *in situ* heating techniques in the STEM, we observed a series of phase transformations within an individual hafnium dioxide nanorod, in real time and with atomic resolution. The phase transition appears to be nucleated at a twin boundary and proceeds through stabilization of discrete domains within the nanorods providing direct evidence that such twin boundaries mediate monoclinic–tetragonal transitions. The reduced transition temperature can be attributed to particle size not just as a result of surface free energy considerations but also in terms of energy dissipation facilitated by twin variants that span the width of the nanowires and that are also engendered as a result of dimensional confinement. Interestingly, rather than the expected martensitic, athermal, transformation, our nanorod underwent a typical nucleation and growth process, to transform from monoclinic to tetragonal hafnia, suggesting that at sufficiently small crystal sizes it may be possible to stabilize phases via kinetic trapping, that in the bulk may not be possible to quench. The propagation of a transformation dislocation one lattice plane at a time (with retention of lattice correspondence across the monoclinic/tetragonal phase boundary) seems to underpin the slow growth of the tetragonal domains allowing for a direct link between atomic resolution imaging and kinetics. On slow cooling the crystal reduces to hafnium metal—likely its thermodynamically stable ground state, due to small crystal size and low oxygen fugacity. The profound fundamental dependence of the phase diagram of this simple binary material upon crystallite size, illustrated by our real-time atomistic study, has wide implications for devising mechanism-informed means for the study and prediction of nanomaterial stability and for the stabilization of metastable structures that are inaccessible in the bulk. Indeed, stabilization of phase-pure tetragonal HfO_2_ below a threshold size of 3.6 nm from direct synthesis based on severe retardation of condensation kinetics suggests that at substantially smaller dimensions strain and size can allow for trapping of this phase^9^. Future work will focus on quenching studies under higher oxygen fugacity (as such measurements become technologically feasible), and an investigation of the transient orthorhombic phase, which is known to be of interest due to its polarity^19^.

## Methods

### Nanorod synthesis

Hafnium(IV) chloride and tri-*n*-octylphosphine oxide (TOPO) were purchased from Strem and used without any further purification. Hafnium(IV) *tert*-butoxide was purchased from Alfa Aesar and used as received. To synthesize the HfO_2_ nanorods equimolar amounts of HfCl_4_ and Hf(O^*t*^But)_4_ were mixed with ca. 10 g of TOPO in a three-neck round bottom flask within an Ar ambient glovebox. The reaction mixture was then transferred and heated under an Ar ambient Schlenk line to ca. 60 °C upon which the TOPO melted and stirring was initiated. Next, the reaction mixture was heated to 340 °C and held at temperature for 2 h. Finally, the reaction mixture was cooled to ca. 60 °C and a solvent/non-solvent washing of acetone and hexanes was performed to remove excess TOPO.

### *In situ* STEM

STEM images were acquired using a Nion UltraSTEM 200 microscope (Oak Ridge National Laboratory) operated at 200 kV. Conventional imaging was performed to characterize the growth direction and crystal phase of the nanorods before heating was performed. Protochips e-chips were used for the *in situ* heating experiments. As-synthesized HfO_2_ nanorod powder was ground and dispersed in hexane and dropcast onto the e-chips. The e-chips were contacted to a Nion electrical cartridge. Protochips Aduro heating software and Keithley power supply were used to resistively heat the chips in the UltraSTEM 200 microscope.

### Image analysis

CrystalMaker software was used to produce simulated single crystal and powder diffraction patterns which were used for phase analysis of the HfO_2_ nanorods. Model structures were built using crystal structure information for the monoclinic^48^, tetragonal^15^, cubic^49^ and orthorhombic^50^ phases found in the literature. A subset of representative images was selected from the images acquired during nanorod heating and cooling in the STEM. For each frame selected, an FFT was acquired in Digital Micrograph. To identify the phases present in each region of the nanorod, analysis of the FFTs was performed for each frame of the movie, as depicted in Figs 3 and 4. This analysis consisted of (1) identifying the component patterns which make up the FFT, (2) indexing and thereby identifying each component and (3) masking each component in the FFT in turn, and performing an IFFT to regenerate only those regions of the rod comprising that component (as further illustrated in Supplementary Methods). Supplementary Figs 10–12 and Supplementary Tables 1 and 2 aid in demonstrating how the FFT patterns were identified and indexed. The IFFTs were false coloured using Adobe Photoshop CS6 and overlaid on the respective unedited nanorod micrograph. QSTEM V2.4 was used to run the STEM simulation. A slab of 2*x* by 3*y* by 3*z* unit cells was used to calculate a simulated HAADF image as viewed down the [011] monoclinic zone. Twenty sub-slices per slab were calculated. The simulation was run for 200 kV accelerating voltage and a detector with inner angle 70 mrad and outer angle of 200 mrad.

### DFT calculations

First-principle calculations were performed within the framework of DFT, as implemented in the Vienna *ab initio* simulation package, applying the generalized gradient approximation using the Perdew–Burke–Ernzerhof functional^51^. The electronic configurations of the relevant elements were realized using the projector augmented wave pseudo-potentials formalism. Brillouin zone integrations were performed using a Monkhorst-Pack mesh with at least 5000k points per Brillouin zone or cell. Full relaxations were realized by using the Methfessel–Paxton smearing method of order one^52^, and self-consistent static calculations were carried out with the tetrahedron smearing method with Blochl corrections^53^. A cutoff energy of 533 eV was used and spin polarizations were accounted for as well. Convergence of the electronic structure was assumed, when changes between two consecutive steps fell below 10^−7^ eV.

Energy landscape calculations were carried out in accordance with the model outlined by Luo *et al*.^54^ by assuming small lattice distortions and uniform transformation of lattice vectors. The contributions of vibrational and electronic degrees of freedom to the total free energy of the system were considered to calculate the finite temperature free energy. The vibrational contributions to the free energy—derived from the phonon DOS—were calculated using the harmonic approximation, which assumes small atomic oscillations about the equilibrium value. In this work, we used the supercell method—as implemented in the ATAT package^55^—to determine the phonon structure of the systems studied. The force-constant tensor that relates the interatomic forces to the atomic displacements from equilibrium was obtained from DFT calculations. The force constant tensor was then used to determine the dynamical matrix whose diagonalization yields the eigenvalues (frequencies) and eigenvectors (displacements) of the normal modes of oscillation (phonons).

### Data availability

The data that support the findings of this study are available from the corresponding authors (B.G. and S.B.) upon request.

## Additional information

**How to cite this article:** Hudak, B. M. *et al*. Real-time atomistic observation of structural phase transformations in individual hafnia nanorods. *Nat. Commun.* **8,** 15316 doi: 10.1038/ncomms15316 (2017).

**Publisher's note:** Springer Nature remains neutral with regard to jurisdictional claims in published maps and institutional affiliations.

## Supplementary Material

Supplementary Information Supplementary Figures, Supplementary Tables, Supplementary Methods and Supplementary References.

Supplementary Movie 1 Video of the HfO_2_ nanorod shown in Figure 3 annealed at 600 °C for 85 mins in the STEM. Phase transformation from monoclinic to tetragonal HfO_2_ upon heating is visible.

Supplementary Movie 2 Video of the HfO_2_ nanorod shown in Figure 3 cooled from 600 °C to 466 °C over 164 mins. Reduction of the nanorod from hafnia to hafnium metal upon cooling is visible.
