# Supplementary material for: Real-time atomistic observation of structural phase transformations in individual hafnia nanorods
Source: Nat Commun. 2017 May 12;8:15316. doi: 10.1038/ncomms15316 (PMC5437304; doi:10.1038/ncomms15316)
Supplement: Supplementary Information — Supplementary Figures, Supplementary Tables, Supplementary Methods and Supplementary References. [file ncomms15316-s1.pdf]

Supplementary Figures

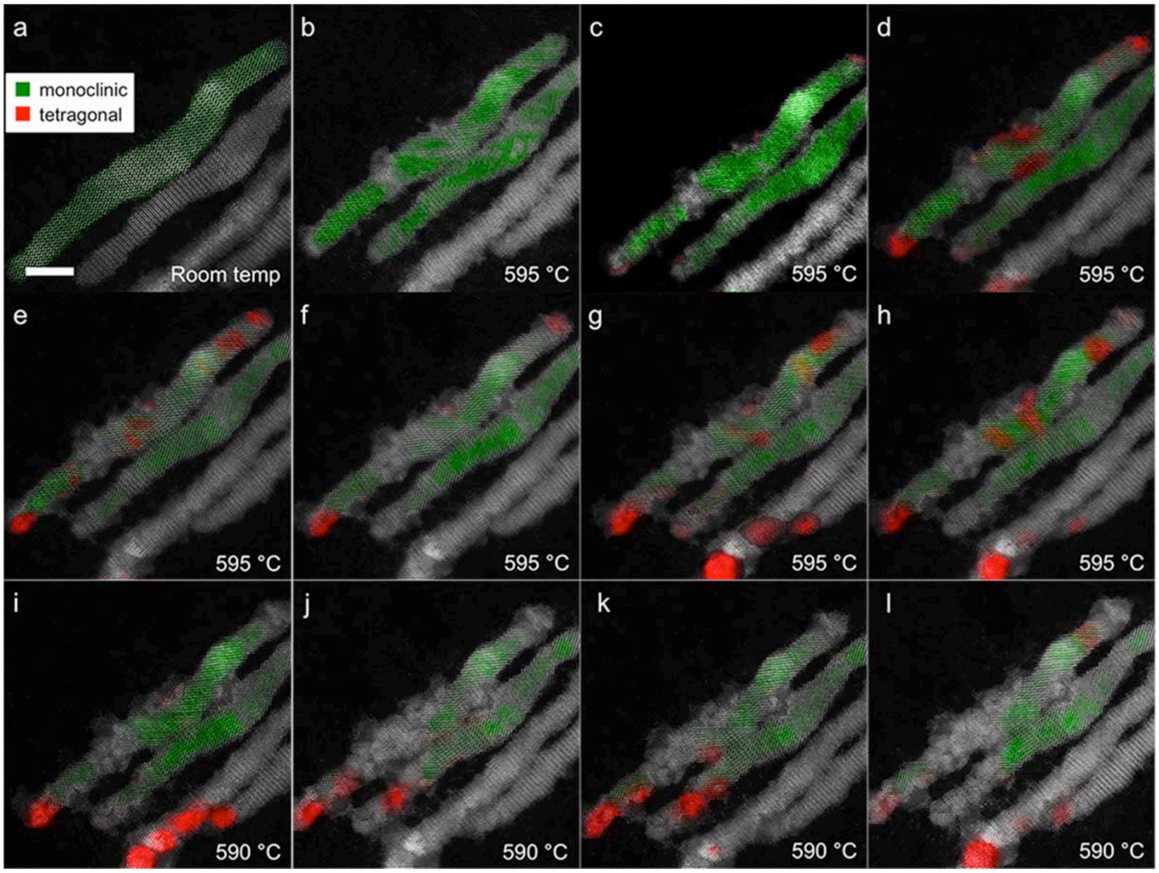

Supplementary Figure 1: *In situ* heating of HfO_2 nanorods. False-colored HAADF frames collected during the heating of a small group of hafnia nanorods. The monoclinic phase is seen to disappear while the tetragonal phase begins to form. The transformation is polycrystalline, and the single crystallites cannot be unambiguously identified, but phase transformation can be directly observed. Scale bar = 5 nm.

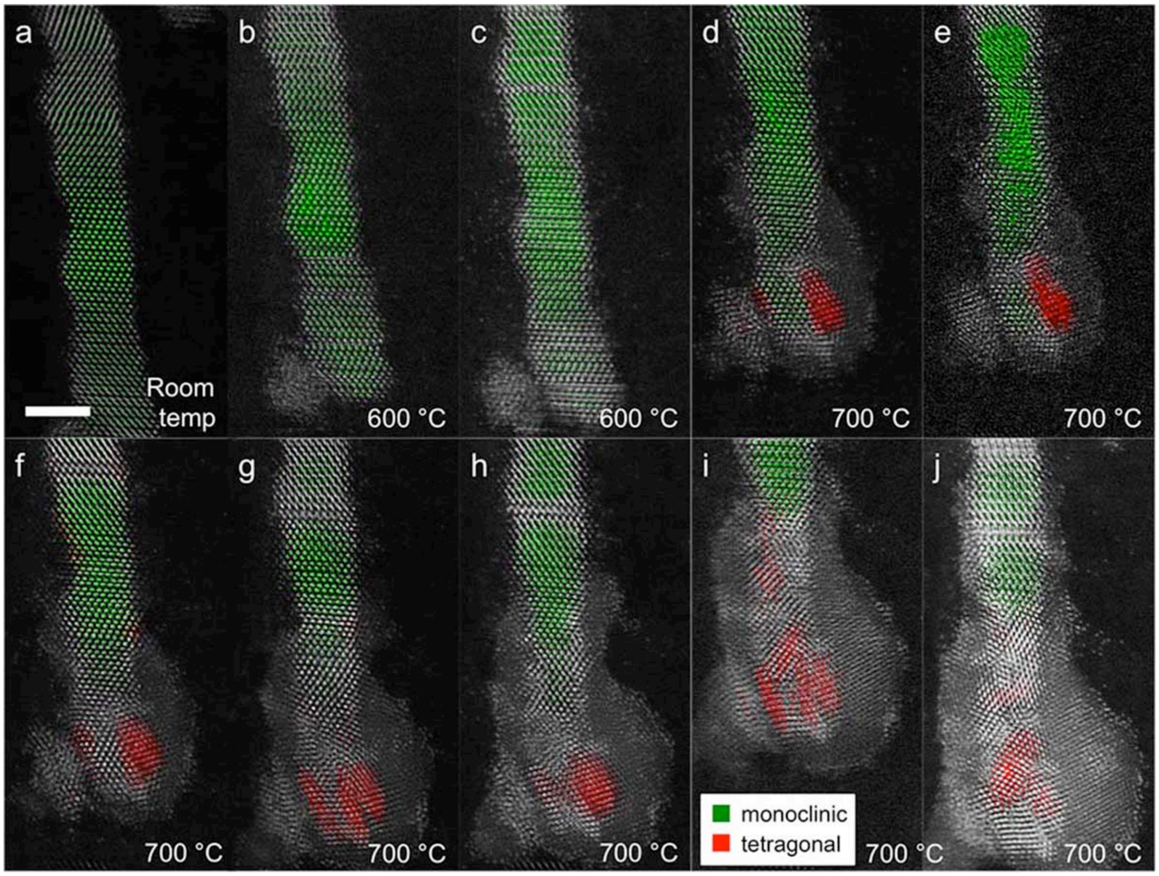

Supplementary Figure 2: *In situ* heating of a single HfO_2 nanorod. False-colored HAADF frames collected during heating of a hafnia nanorod. The wire is seen to undergo a transformation, with the monoclinic phase being transformed into a polycrystalline structure. Some of the crystallites can be identified as the tetragonal phase, but other crystallites are too small to completely identify. Scale bar = 5 nm.

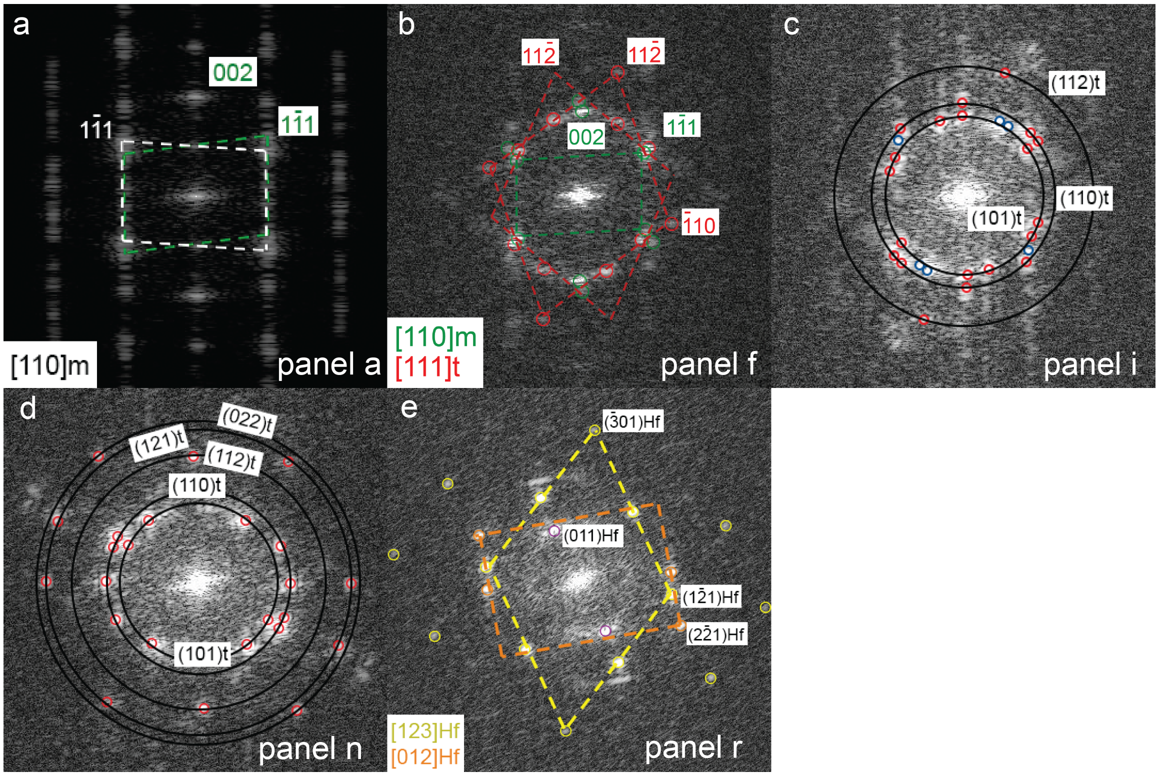

Supplementary Figure 3: Indexing selected frames from Figure 3. Indexed FFTs from Panels a, f, i, n, and r from Fig. 3 of the main text. (a) Panel a: monoclinic HfO_2 in the [110] zone, two twin domains. (b) Panel f: monoclinic HfO_2 in the [110] zone; two orientations of tetragonal HfO_2 in the [111] zone. (c) Panel i: tetragonal HfO_2 spots (red), and unidentified spots (blue). All tetragonal spots are from the [111] zone. (d) Panel n: Tetragonal HfO_2 spots from the [100], [110], and [111] zones. These zones are identified in Supplementary Figure 7. (e) Panel r: Hf metal spots from the [123] and [012] zones.

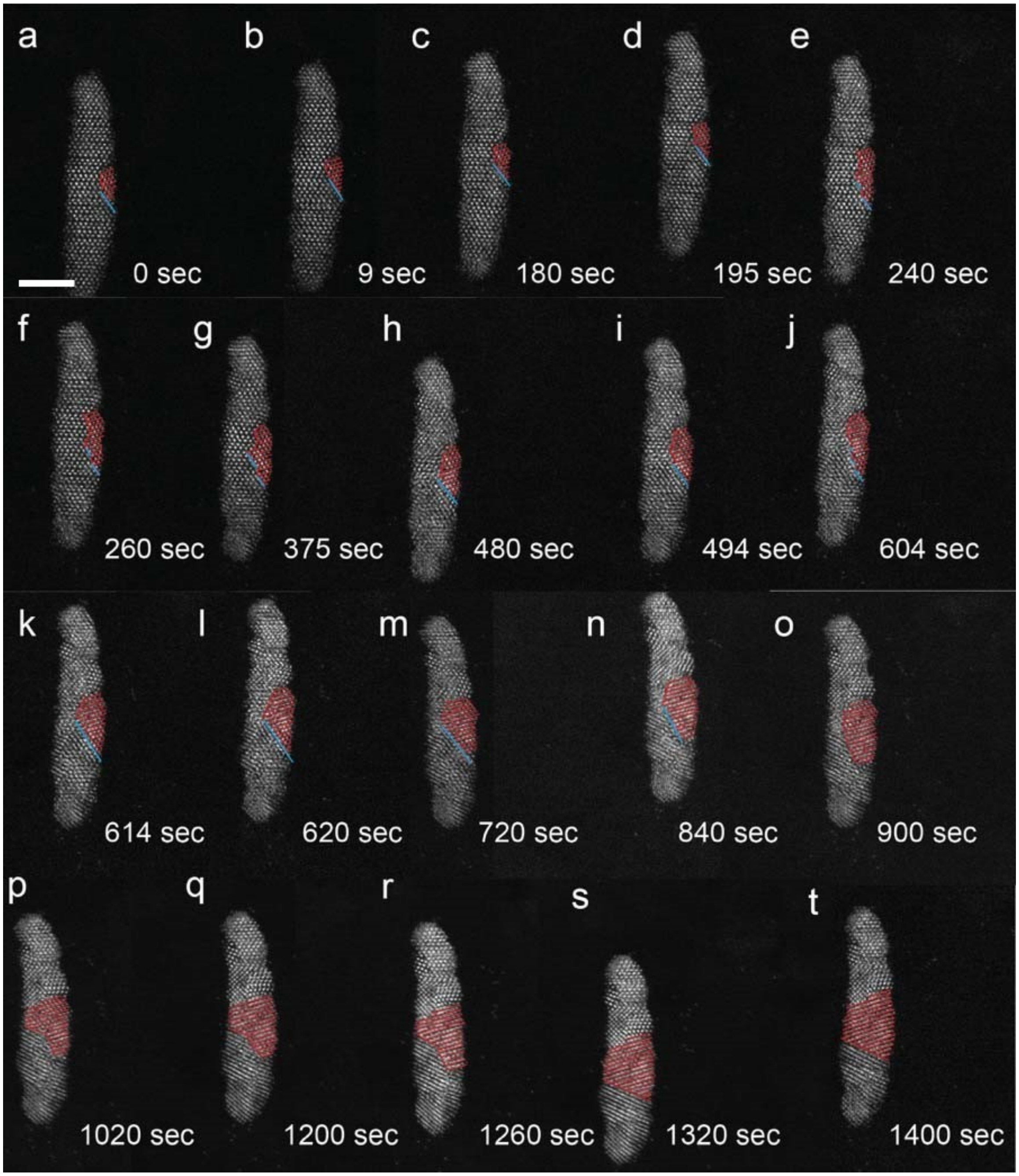

Supplementary Figure 4: Propagation of transformation dislocation. Highlighting the growth of a single domain of tetragonal HfO_2, within the rod presented in Fig. 3 of the main text. The lower boundary of this domain (highlighted in blue) is a monoclinic {111} / tetragonal {011} interface, until ~840 s into domain growth. Scale bar = 5 nm.

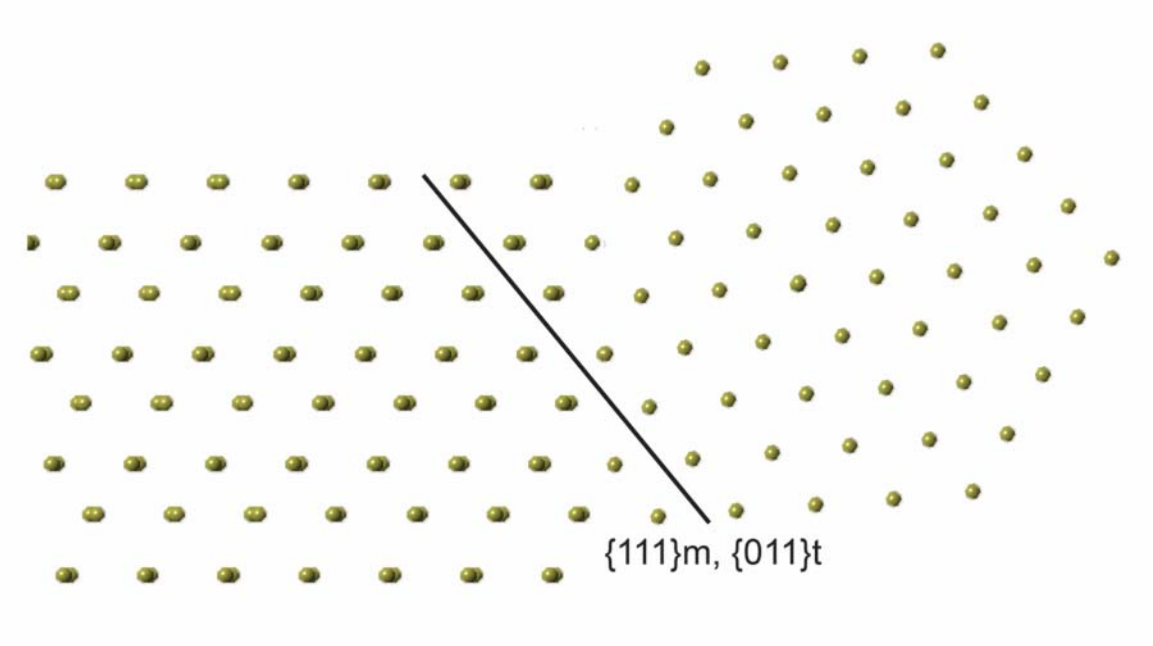

Supplementary Figure 5: Coherent phase boundary. Coherent phase boundary formed by the {111} planes of monoclinic HfO_2 with the {011} planes of tetragonal HfO_2. Oxygen atoms have been omitted from the cartoon for clarity.

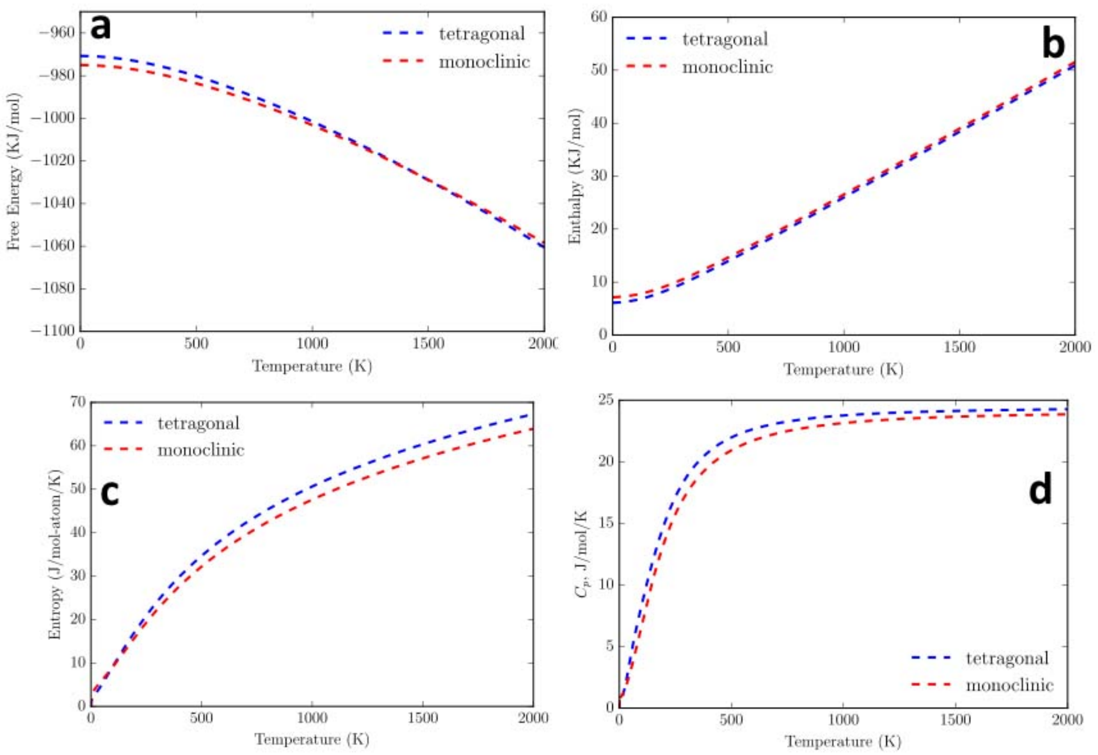

Supplementary Figure 6: Calculated temperature dependencies for the bulk. Finite temperature free energy, enthalpy, entropy and heat capacity calculated for monoclinic and tetragonal phases of HfO_2. (a) Calculated free energy plotted as a function of temperature indicating the stabilization of the tetragonal phase only at higher temperatures. (b) Calculated enthalpy of the monoclinic and tetragonal phases as a function of temperature. (c) Calculated entropy as a function of temperature indicating a substantial increase in the entropy differential with increasing temperature, which results in an increased entropic contribution to the free energy of the phase transition at higher temperatures. (d) specific heat as a function of temperature for the monoclinic and tetragonal phases of HfO_2.

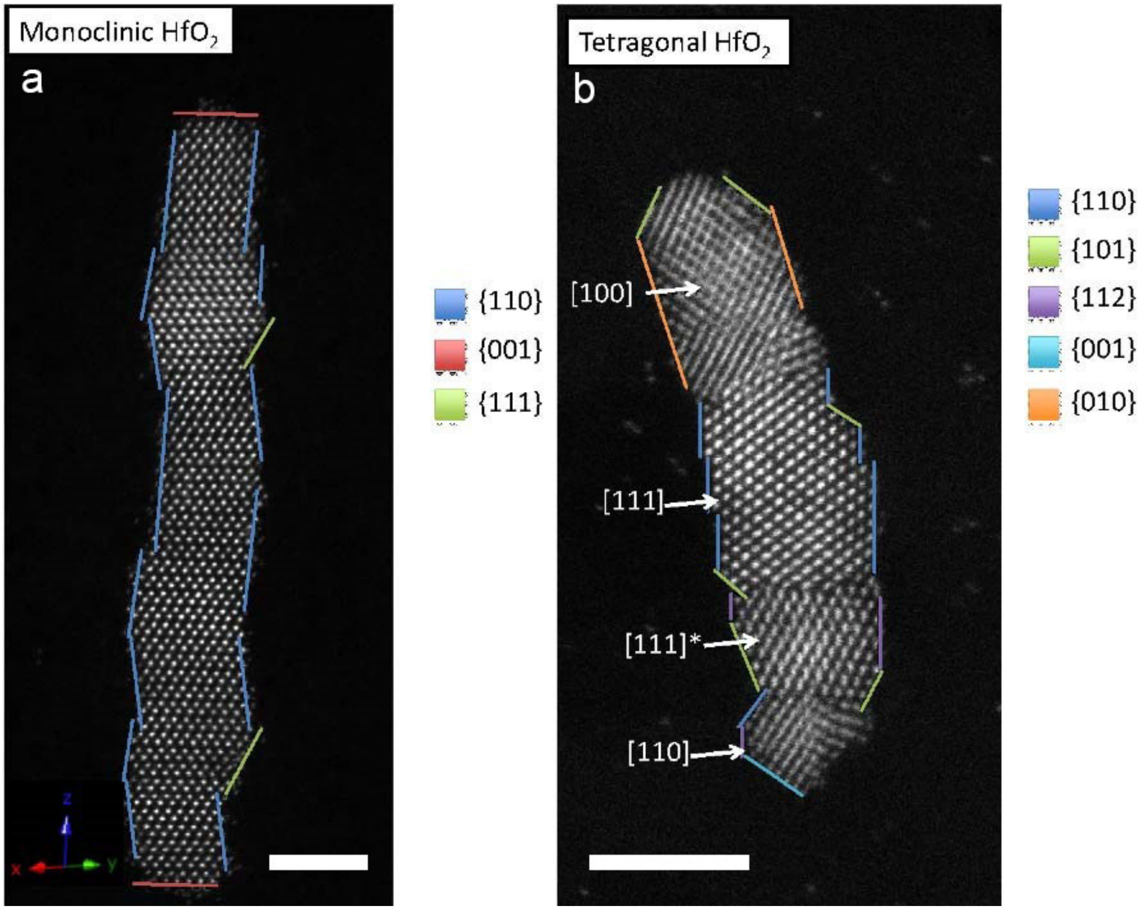

Supplementary Figure 7: Surface planes of monoclinic and tetragonal HfO_2. Identification of surface planes across the phase transformation. (a) The surface planes of monoclinic HfO_2. The side surfaces are {110} planes, with some anomalies arising due to the twin planes. The image was acquired along the [110] zone axis. (b) The surface planes of tetragonal HfO_2. Zone axes are labeled to the left of the nanorod. Scale bars = 5 nm.

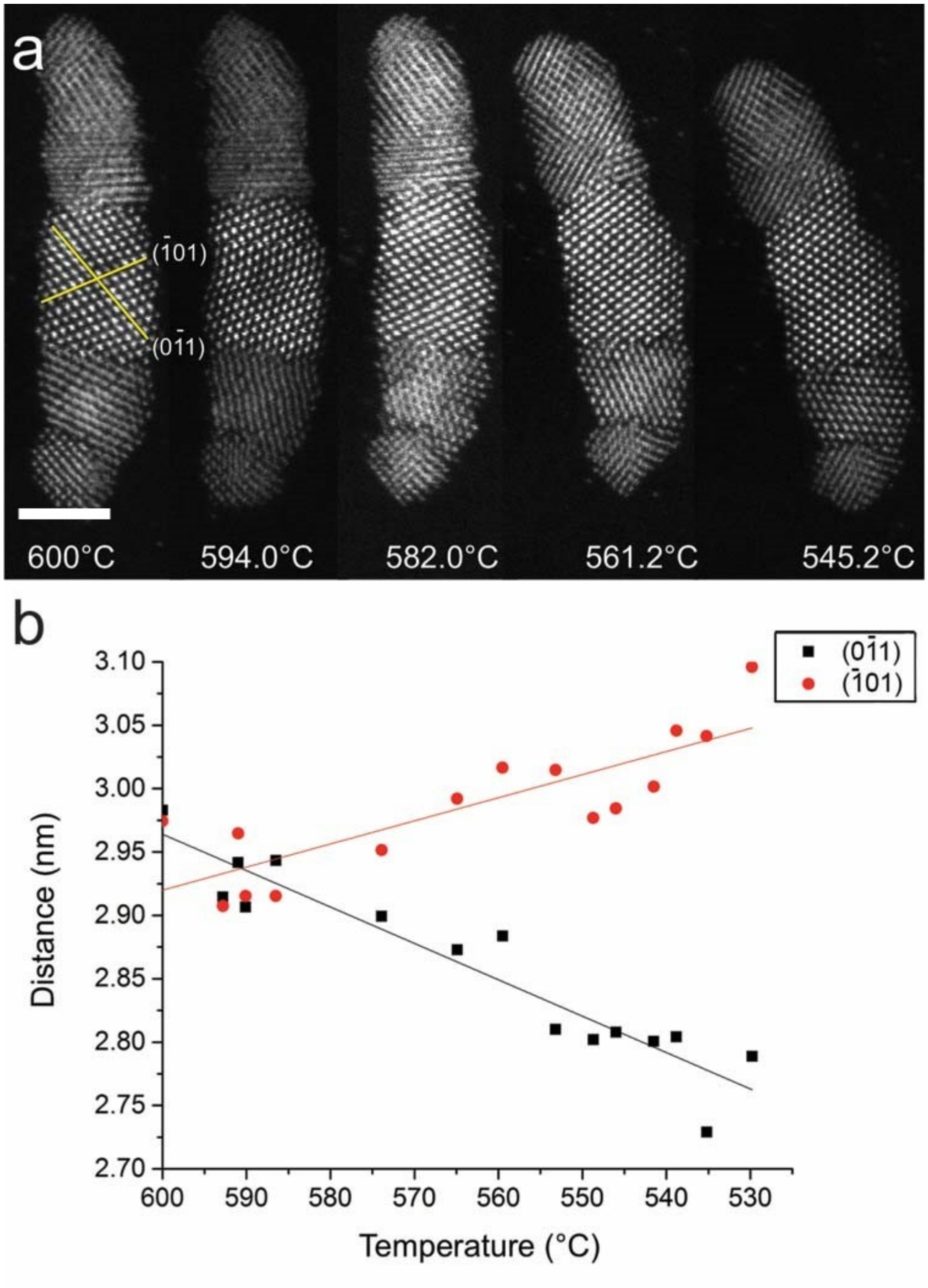

Supplementary Figure 8: Tetragonal hafnia distortion upon cooling. (a) Atomic resolution images of the nanorod with the center segment aligned down the $[111]$ zone axis. The $(0\bar{1}1)$ and $(\bar{1}01)$ planes are marked in the first panel. (b) Plot of *d*-spacing of the $(0\bar{1}1)$ (black squares) and $(\bar{1}01)$ (red dots) planes measure in nm *versus* temperature during cooling. Scale bar = 5 nm.

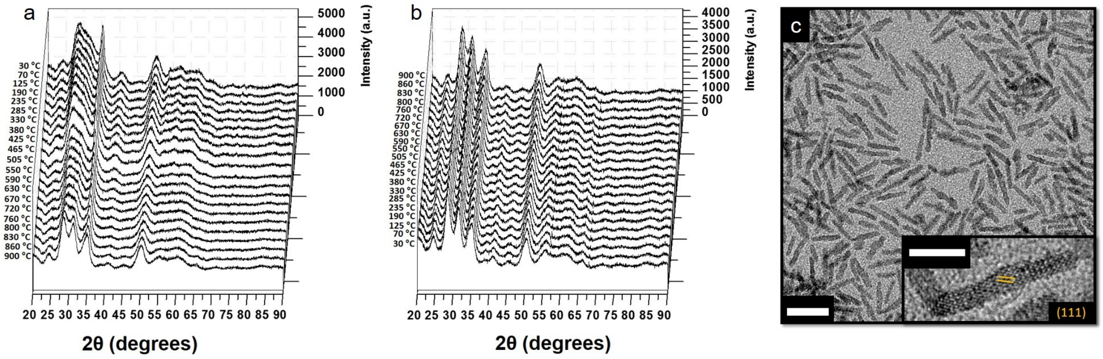

Supplementary Figure 9: Temperature-dependent X-ray diffraction. (a) Evolution of the powder diffraction pattern of HfO_2 nanorods upon annealing from 30 to 900°C. (b) Evolution of the powder diffraction pattern of HfO_2 nanorods upon cooling from 900 to 30°C. Reduction to metallic Hf is not observed outside of the reducing environment of the electron microscope. (c) TEM image of the HfO_2 nanorods used for the temperature-variant XRD experiments. Scale bar = 20 nm. The inset shows a lattice-resolved HRTEM image of an individual nanowire. Scale bar = 5 nm.

Supplementary Methods

Detailed Description of Phase Identification Procedure

In order to identify the phases present in each region of the nanorod, analysis of the fast Fourier transforms (FFTs) was performed for each frame of the movie, as depicted in Figures 3 and 4. This analysis consisted of (1) identifying the component patterns which make up the FFT, (2) indexing and thereby identifying each component, and (3) masking each component in the FFT in turn, and performing an inverse FFT (IFFT) to regenerate only those regions of the rod comprising that component.

Step 1. First, an FFT is acquired from the entire frame, producing (in some cases) a complex convolution of FFTs from the various regions of the wire. Next, FFTs are acquired from smaller regions of the rod, to identify the spots corresponding to distinct regions with only one crystal phase and orientation. A sufficient number of component FFTs from all regions of the rod are identified, to account for every spot in the full FFT. Using the full FFT, the spots from any one component FFT are masked, and an IFFT is performed, generating an image of only those region(s) of the rod with this particular crystal phase and orientation. (It should be noted that, consistent with this approach, if a non-systematic array of spots from the full FFT are masked rather than those identified as coming from a single crystallite component, the IFFT does not generate an image of a single region of the rod, but instead a convoluted image of the entire rod.) An example of this process from Figure 3n is shown below.

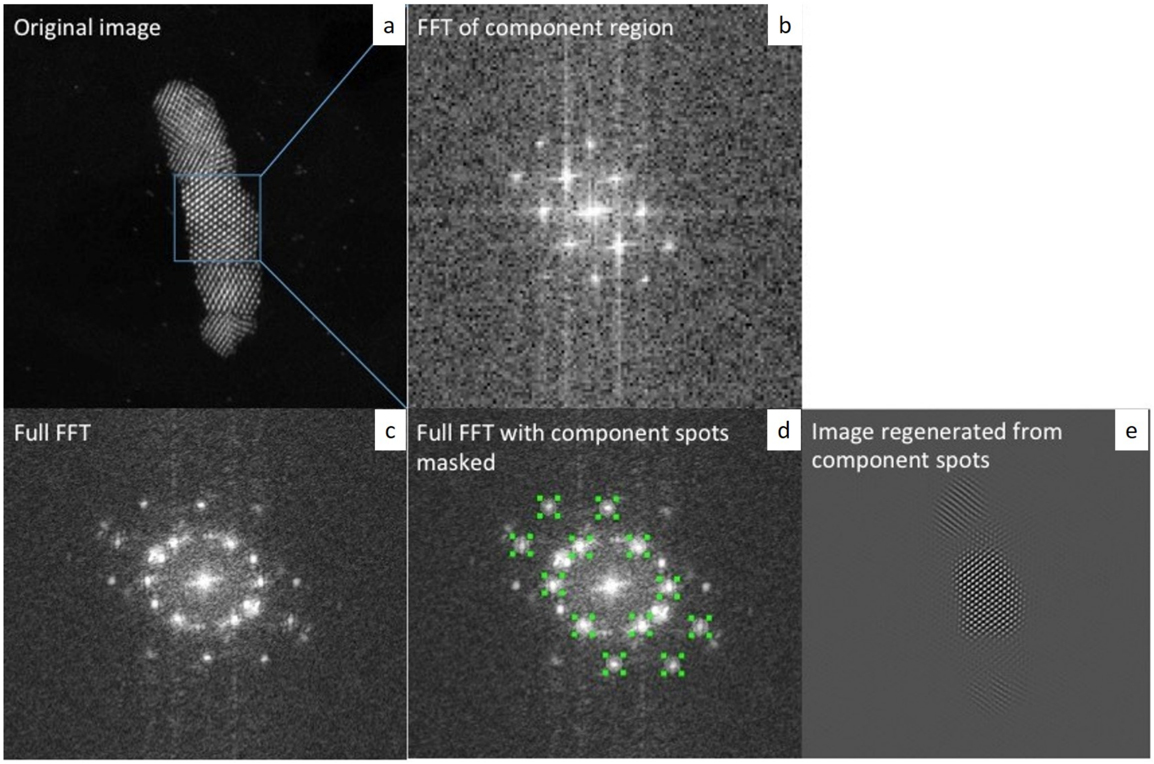

Supplementary Figure 10: Steps taken to identify component FFTs. (a) Original HAADF image of a nanorod (Figure 3n). (b) FFT of a square region marked in (a). (c) FFT of the entire nanorod. (d) Component spots from one crystal phase are masked in the full FFT. (e) Image regenerated by performing an IFFT of the masked spots.

Step 2. Having identified each component from the full FFT, the next step is to index each component FFT, to identify the phase and orientation of the crystallite responsible for those spots. Using Figure 3f as an example (Supplementary Figure 11), shown in Supplementary Table 1 are the measured distances from the origin, and angles, of each FFT spot from a single component, along with the monoclinic, tetragonal, and cubic HfO_2 *d*-spacings which most closely fit the experimentally measured values, and the corresponding angles of those plane-normals with respect to one another. For the example shown, four potential phase and orientation combinations are identified, and three of the four (tetragonal [111] zone, tetragonal [100] zone, and cubic [110] zone) have patterns with the same symmetry as the experimental data.

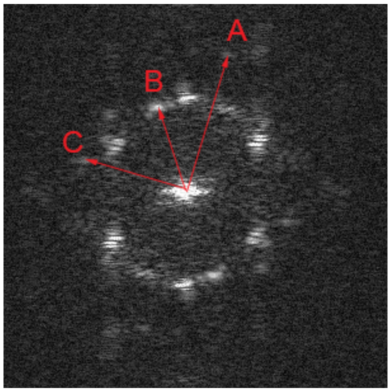

Supplementary Figure 11: Full FFT of Figure 3f. The FFT contains multiple crystallites. One crystallite has been identified. The arrows marked A, B, and C indicate spots belonging to that one crystallite and are used to identify the crystallite phase.

	**Experimental (pix)**	**Mono[1] (Å^{-1})**	**Plane**	**Tet [111] (Å^{-1})**	**Plane**	**Tet [100] (Å^{-1})**	**Plane**	**Cubic[2] [110] (Å^{-1})**	**Plane**
**Distance A**	112.71	0.558	{$\bar{1}$22}	0.558	{112}	0.562	{020}	0.554	{220}
		0.554	{220}						
		0.565	{$\bar{2}$21}						
**Distance B**	69.58	0.354	{111}	0.342	{101}	0.342	{101}	0.339	{111}
**Distance C**	80.39	0.396	{200}	0.397	{110}	0.391	{002}	0.391	{002}
**Angle AB**	35.5			35.46		34.86		35.26	
**Angle AC**	90.0			90.0		90.0		90.0	
**Angle BC**	54.5			54.54		55.14		54.74	

Supplementary Table 1: Measured angles and distances. The distance to and angles between Supplementary Figure 11 spots A, B, and C were measured from the origin. The *d*-spacing and angles of the monoclinic, tetragonal, and cubic phases which most closely fit the experimental data were identified.

Having narrowed down the assignment to these three candidates, a comparison of the fit to the experimental data is made, revealing (in this example) that the tetragonal [111] zone is the closest fit to the experimental data. Supplementary Table 2 summarizes the comparison of experimental data with the three crystal phases that most closely resemble the experimental structure.

	Experimental	Mono	Tet [111]	Tet [100]	Cubic [110]
**Distance A/B**	1.620	1.576	1.632	1.643	1.634
**Distance A/C**	1.402	1.409	1.406	1.437	1.417
**Distance B/C**	0.8655	0.894	0.861	0.875	0.867
**% Err ∠AB**		--	0.113	1.84	0.681
**% Err ∠AC**		--	0.000	0.000	0.000
**% Err ∠BC**		--	0.0733	1.16	0.448
**% Err Dis A/B**		2.765	0.7181	1.425	0.8785
**% Err Dis A/C**		0.5004	0.2491	2.456	1.047
**% Err Dis B/C**		3.178	0.4724	1.046	0.1704

Supplementary Table 2: Comparison of experimental data to crystal structures. The percent error between the experimental data and the calculated crystal structure data for the crystal phases that most closely matched the experimental data were calculated. Based on this, a conclusion can be made as to which phase the identified FFT in Supplementary Figure 11 belongs.

Comparison of the calculated selected area electron diffraction patterns, shown in Supplementary Figure 12, (using SingleCrystal software from the CrystalMaker software suite) with the experimental data further allows the elimination of tetragonal [100] and cubic [110] since these patterns contain additional peaks which are not present in the full FFT.

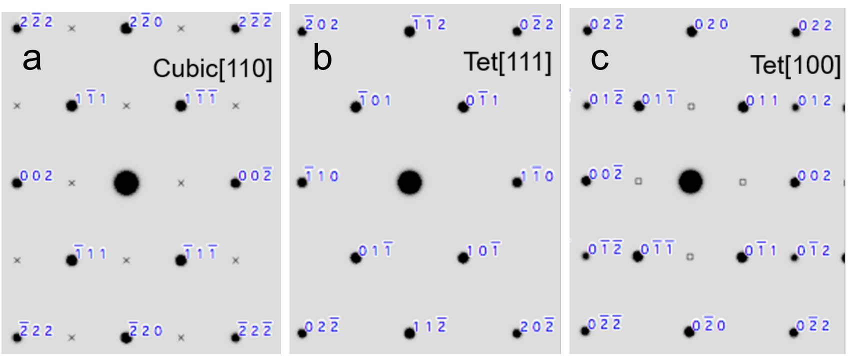

Supplementary Figure 12: Calculated selected area electron diffraction patterns. Selected area electron diffraction patterns of the crystal phases that most closely match the experimental data were calculated using SingleCrystal software from the CrystalMaker software suite. The calculated patterns were used to identify the crystal phase and orientation indicated in the experimental data. (a) Cubic [100] diffraction pattern. (b) Tetragonal [111] diffraction pattern. (c) Tetragonal [100] diffraction pattern.

Step 3. So identified, the spots from the component FFT are masked, and used to perform an IFFT, generating an image of only those regions of the rod with the phase and orientation of the indexed pattern. In this way, each region of the rod, in each timeframe of the movie, is identified, as depicted in Figure 3.

Supplementary References

1. Ruh, R. & Corfield, P. W. R. Crystal Structure of Monoclinic Hafnia and Comparison with Monoclinic Zirconia. *J. Am. Ceram. Soc.* **53,** 126–129 (1970).
2. R. W. G. Wyckoff. *Crystal Structures*. **1,** (Interscience Publishers, 1963).
